# Blood donor incentives: A step forward or backward

**DOI:** 10.4103/0973-6247.59385

**Published:** 2010-01

**Authors:** Hassan Abolghasemi, Nasim S. Hosseini-Divkalayi, Fariba Seighali

**Affiliations:** *Department of International Affairs, International Law MA graduate, Iranian Blood Transfusion Organization, Tehran, Iran*; 1 *Pediatric Gastroenterologist, Iranian Blood Transfusion Organization Research Center, Iran*

**Keywords:** Blood donor, voluntary non-remunerated blood donation, paid donation, incentive, blood safety

## Abstract

Dramatic increase in blood usage and critical seasonal blood shortages are faced by various countries. Countries which previously reached 100% voluntary nonremunerated donation have been led to offer different kinds of incentives to recruit blood donors and meet their blood demands. In some cases, these incentives are considered monetary and are in complete contrast with International standards like World Health Organization (WHO). It seems that attitudes toward sole dependency on nonremunerated voluntary blood donation have been changed in recent years and experts in some developed countries are reevaluating partial reliance on paid donation. On the other hand, besides the effects of such incentives on blood safety, several economic and psychological studies have shown that incentives have discouraging effects on pro-social behaviors like blood donation and will reduce the number of blood donors in long term. With regard to the results of such studies, it seems that implementing incentive-based blood donor recruitment programs to meet blood requirements by some countries is becoming a challenge for blood banks.

## Introduction

In 1975, World Health Organization (WHO) adopted a resolution encouraging countries to promote the development of national blood services based on voluntary nonremunerated blood donation.[1] Also, WHO set a goal for all blood donations to be collected from unpaid volunteer donors by 2020.[2] This issue has also been highlighted in Melbourne declaration composed by an international group of experts and participants of 2009 World Blood Donor Day in Australia, urging all countries to achieve 100% voluntary nonremunerated donations by 2020.[3] As most developing countries are trying to reach 100% voluntary nonremunerated blood donation by applying WHO standards, in some countries such as United States offering material incentives to recruit blood donors is becoming a common way to meet blood demands. But is recruiting blood donors, by material incentives, an effective and safe way to increase the blood supply? This paper intends to assess the trend of blood donation pattern in recent 50 years and investigate the challenges resulted from offering incentives on blood safety and blood donors contributions. Information of this article was drawn from more than 20 epidemiological, economic, and psychological studies assessing the reasons, types, and effects of introducing incentives to blood donors in the last four decades.

## Trend of Blood Donation Pattern in Recent 50 Years

Two studies in the late 1959 showed a higher incidence of post-transfusion hepatitis in paid and professional donors.[4,5] In the early 1970s, all organizations participating in blood transfusion procedures in United States issued position statements supporting the concept of voluntary donation.[6] In 1978, the FDA requested that all blood and blood products to be labeled as “paid” or “volunteer.”[7] In accordance with this trend, voluntary blood donation was promoted in European countries too. This kind of donation has been accepted for over 50 years in France, Luxembourg, Northern Ireland, and also it was introduced in Spain in the 1980s, Italy, Portugal, Greece, and Estonia in the 1990s.[8]

In 1990s, the requests for blood and blood products increased dramatically. The margin between blood supply and demand in United States decreased from 13.8% in 1989 to 9.1% in 1999.[9] Factors such as advances in surgeries and cancer treatment, tightening the blood donation criteria, seasonal shortages, and aging of the blood donor populations have played an important role in increasing blood demands.[10,11] According to the latest survey, the number of blood transfusions in United States has increased from 1.1 million in 1997 to 2.7 in 2007 (about 140% increase in demands for blood transfusion).[12]

Pablo Rodriguez Del Pozo in a study published in 1994 stated that relying solely on voluntary donation means putting up with blood and plasma shortages. Thus, he believes that the most economically rational and socially constructive model seems to be a system in which volunteerism is the rule and payment the exception. He believed that due to disadvantages of all-volunteer system including promoting the risk of illegal market and the shortage of blood derivatives, it is illogical to relay completely on voluntary blood donation.[13]

The mentioned factors resulted in beginning the debates over voluntarism versus paid donation (Research and Progress session at the 2001 American Association of Blood Banks (AABB) annual meeting). Today, some experts believe that blood donor attitudes toward compensatory incentives may have changed in the last 20 years and all-volunteer system should be reevaluated.[14] Some believe that the sole altruism incentive is not enough to meet all blood requirements.[15] Jay Pennington says “nothing is free in the life”. This statement is the beginning sentence of his article, supporting material incentives, which has been published in AABB website. He believes that elements of truth represented in this adage may be apparent even in the altruistic setting of blood donation.[16]

## Types of Material Incentives

To meet blood requirements and overcome the shortages, some blood banks try to recruit blood donors by offering different kinds of material incentives. In some countries such as United States, blood donors are openly enticing by monetary incentives.

### Incentives in United State of America

Donors in the United States are often recruited by widespread use of rewards or incentives for donating blood, which may include t-shirts, event tickets, or opportunity drawings for televisions and expensive cars. In this context, American Red Cross in September 2008 started a campaign called “Give a Little, Buy a Lot” aimed to increase blood and platelet donations during the holidays. In this campaign, donors have a chance to win a $1000 gift card for shopping.[17]

This is not the first time that American Red Cross or other blood banks in United States offer material and monetary incentives to increase the number of blood donors. In 2008, a judge in South Florida gave a16-year-old boy a choice to donate a pint of blood or pay fine for under-age smoking at the mall. Defendants who appear before shutter can lop off as much as $75 from their fines or receive credit for community service if they donate blood. The option is available to traffic offenders whose violations range from expired vehicle tags to unintentionally killing someone in a car accident.[18]

The Compliance Policy Guide written by the FDA in 2003 describes how material incentives except monetary ones would require the blood to have the “paid donor” classification statement. In accordance with this Policy Guide, the donation is considered paid if the incentives are transferable, refundable, or redeemable for cash and a market exists for the incentives. For example, Ticket vouchers for symphony or opera performances may require a “paid donor” label to be placed on the products if an accessible market exists for the tickets and they are transferable. The regulation specifies benefits that would not require the “paid donor” classification statement, as long as the benefits are not readily convertible to cash. These benefits are (1) time off from work, (2) membership in blood assurance programs, and (3) cancellation of nonreplacement fees.[19]

In a study conducted in 2001, it was found that 56% of American blood donors received some kind of incentive, including items of appreciation (26.8%), paid time off work (22.4%), blood credits (8.1%), and cash (0.2%).[20]

### Incentives in European countries

In a study conducted in 2001 in 17 European countries which analyzed the national regulations concerning blood safety across Europe, it was shown that only five countries including Finland, Republic of Yugoslavia, Slovenia, Spain, and the United Kingdom have exclusively voluntary, nonremunerated blood donation and receive no particular incentive (apart from light refreshment following donation). The Czech Republic, Greece, Italy, Macedonia, Romania, Croatia, and possibly France report that their donors are voluntary, nonremunerated, but all (Greece, Macedonia, and Romania) or part of them (Croatia, Czech Republic, and Italy) receive some sort of incentive.[21]

These incentives range from one (or more) day off work and travel expenses to tax reliefs and other material gifts. In the Czech Republic, an employee can have free time only for blood donation and recovery. In Slovenia, an employee may be absent from work on the day of donation with compensation by the employer, payable by the health insurance. In France, offering the monetary incentives to blood donors, directly or indirectly, is forbidden. But reimbursement to blood donors for travel expenses is authorized and remuneration for time off work paid by the employer to the donor may be maintained during the time spent for the donation without being considered as a payment in so far as the duration of the absence does not exceed the time necessary for the displacement between the working place and the donation place.[8]

Dramatic decrease in the number of voluntary donors and seasonal shortages forces some countries to meet their blood requirements by offer monetary incentives. In the Czech Republic, a blood donor may receive tax relief (€10 per donation; maximum€50-70 annually as per individual tax rate). The number of blood donors requesting “tax relief” is estimated at 60% as many donations are from people not paying taxes (e.g., students).[8] In August and September 2008, Rome residents received a special voucher which can be exchanged for two free tickets to city museums. First-time donors also received two free tickets for boat service. The initiative is part of the annual summer campaign to encourage people to give blood at a time of year when donations typically drop by 40%.[22]

## Incentives: Encouraging or Discouraging?

There is a serious concern over using incentives in blood donations even on a temporary basis. That concern is based on the findings that using incentives may attract at-risk donors, and worse undermine the motivation to donate blood.

Some studies in psychology and economics have found that material incentive discourages prosocial behaviors and causes decrease in blood supply.

In this text, we review the studies that assessed the challenges of incentives on blood safety and blood donor's contributions.

### Effect of incentives on the number of blood donors

Using incentives for blood donation may undermine the altruistic motivation to donate blood. This concern has always existed after Titmuss study in 1971. He believed that commercializing the altruistic setting in blood donation has crowding-out effect on the number of blood donors.[23] Since then, several economic and psychological studies have shown the same results and proved that incentives have negative effects on prosocial behaviors like blood donation.[24,26]

In a study conducted by Benabou and Tirole in 2006, it was found that people refuse to enter in transactions that seem to have economic benefit for them, but which they judge to be insulting to their dignity.[25] According to another study, the reason for contributing people in public prosocial activities like blood donation is to be well-regarded by others. But with extrinsic incentives, the signal of a prosocial act gets diluted. So individuals may be discouraged to take part in such activities.[26]

Sliwka proposes a model in which there are three kinds of people: Selfish ones, prosocial ones, and conformists. These “conformists” have social preferences if they believe that sufficiently many of the others do too. Offering incentives can signal to conformists that there are more selfish people in society, leading them to also behave in a selfish manner not prosocially.[27] In a study conducted by Ellingsen and Johannesson in 2008, it was shown that the counterparty motives are important for individuals who take part in an activity. Individuals are willing to act prosocially only if they perceive the counterparty as nonselfish. In other words, using incentives may signal the blood donors that blood banks pursues selfish goals, which then blood donors are less desired to act in a way that enhances the other party's payoff and undermine their contribution.[28]

The results of one study in 2003 evaluated the attitudes toward blood donation incentives in United States found that donors at schools or universities and at military sites who are mostly young donors were more encouraged by incentives than donors at other donation sites. In contrast, donors at religious sites who tended to be older were often least likely to find incentives attractive. Although donors would be encouraged to return if offered cholesterol screening (61%), blood credits (61%), and, if male, PSA screening (73%), their findings also indicate that offering cash to blood donors may be detrimental to blood availability by discouraging about 7% current volunteer donors from returning. Also it was found that the highest level of discouragement was obtained for compensatory-type incentives (up to 9% of all donors for lottery or raffle ticket).[14]

In 2008, Mellstrom and Johannesson decided to test the Titmuss hypothesis among Swedish blood donors. In this research, one group was given an opportunity to donate blood without offering any incentives, second group received a payment of $7, and the third group was free to choose between a $7 payment or donating $7 to charity. The results differ significantly between men and women. The supply of blood donors is not significantly different among the three experimental men groups. For women, there is a significant crowding-out effect. The supply of blood donors decreased by almost half when a monetary payment is introduced.[29]

However, two recent studies showed that using selective incentives with observing some conditions has positive effect on donors' contribution. In a large-scale field experiment lasting 3 months and involving more than 10,000 previous donors, two types of incentive were examined: A lottery ticket and a free cholesterol test. Although, lottery tickets significantly increased donations in particular among less motivated donors, the cholesterol test had no impact on blood donations.[30]

However, it should be noted that in this study, all incentives were given privately; consequently there was not any negative public image against blood donors.

In another study carried out in Italy, the effect of two kinds of incentives including one paid day off work and symbolic rewards (“medals”) with social recognition value but no economic value to repeat donors were assessed. They showed that the day-off incentive leads donors who are employees to make, on average, one extra donation per year. As for the symbolic rewards, they also appeared to increase donation frequency, but only when the prizes are awarded publicly and the recipient's names are published in the local newspaper.[15] So as we mentioned before, donors concern about their public image when they donate blood; they want to be regarded by others as prosocial individuals even when they accept incentives. These two studies suggested that using selective incentives (not cash) can increase the number of blood donors during particular occasions (not on permanent basis) like seasonal shortages. But it is worth mentioning that both studies were conducted among lapsed donors whose concern over their blood safety is much less than the first-time donors.

### Effect of incentives on blood safety

The effects of offering monetary incentives on blood safety of donors have been evaluated in numerous studies. In 1998, Eastlund in a review article assessing 26 previous researches concluded that infectious diseases among donors who are recruited by monetary incentives are more prevalent.[31] A survey conducted by Retrovirus Epidemiology Donor Study (REDS) proved that donors who reported being encouraged to donate by cash incentive were 1.6 times more likely to be at risk for infectious diseases (OR = 1.56; 95% CI, 1.05-2.35).[33] Another study was carried out by European researches in 2002 to review studies published between 1968 and 2001, which evaluated a possible trend of change in the relative risk for infectious disease markers between paid and unpaid blood and plasma donors in the mentioned period. According to this review, there is no significant trend to indicate that relative risks between paid and unpaid donors from 28 published data sets, have decreased over time.[32] Also, promoting voluntary nonremunerated blood donation is the safest way to prevent transmitting new blood borne infectious disease.

Two comparative studies indicated that in 1996 the newly discovered GBV-C virus was found to be considerably more prevalent in paid donors.[32] Also in these cases, there is a huge concern that incentives may affect the safety of the blood supply by attracting at-risk donors who may hide some risky behaviors at the time of donor screening to obtain the incentive. The results of one study conducted in 2001 reported that donors who would be encouraged by incentives tended to have a higher prevalence of unreported deferrable risks. Repeat whole blood donors who would be encouraged to donate by cash and tickets to an event were 75% and 70% more likely to have an Unreported Deferrable Risk (UDR) than donors who would be discouraged or indifferent to these incentives, respectively.[33] Although offering monetary incentives to blood donors has been proved to have negative effect on blood safety, some nonmonetary incentives such as medical tests would probably be well received by donors without detrimentally affecting blood safety.[13,34]

## Discussion

On the basis of our findings, in some countries attitudes toward covering blood requirements using totally voluntary nonremunerated blood donation may have changed during the recent years and offering different kinds of incentives to blood donors is becoming a common way to meet blood demands. Although some findings indicate that using nonmonetary incentives may help increase the number of lapsed blood donors, shifting to permanent incentives may make donors consider blood banks as a nonaltruistic service and think that they pursue selfish goals; then blood donors will be less desired to act in a way that enhances the other party's benefit and undermine their contribution.

Several studies especially economic and psychological ones indicated that offering incentives to blood donors has negative effect on blood donors' contributions. Regular donors who primarily donate for altruistic reasons may be discouraged to return by incentives. In addition, there is still a huge concern over the blood safety of such donors. Latest studies continue to indicate this finding. Although the safety of blood screening tests has been improved in recent year, as paid donors tend to donate during the “window period”, the risk of transmitting infectious diseases during the “window period” has not been completely eliminated. Also, the main concern rises when developing countries with nonadvanced technologies try to follow this trend and dilute their efforts to increase the number of voluntary nonremunerated blood donors. While saving the patient's lives is the highest priority for blood banks, recruiting donors by material incentives may promote the wrong culture of paid donation and undermine the altruistic setting in blood donation.

Our results indicate that offering money or cash-equivalent incentives (such as tickets to an event) may have a negative effect on blood safety and blood donor contribution. Regarding the recent downward trend in blood collection and increasing demands for blood transfusions, our evidence suggests that selective nonmonetary incentives can be used among lapsed donors temporarily when shortages occur. These findings may assist blood banks to revise and optimize their blood donor recruitment programs and to adopt a long-term comprehensive policy with regard to consequences of offering incentives to blood donors.
